# Therapy Culture for the Business Class: Exploring How CEO Peer Groups Make and Legitimate Elite Cohesion

**DOI:** 10.1111/1468-4446.70044

**Published:** 2025-10-22

**Authors:** Katie Higgins

**Affiliations:** ^1^ School of Sociological Studies Politics and International Relations University of Sheffield Sheffield UK

## Abstract

In the current context of extreme economic inequality and rising concentrations of income and wealth at the top, the social processes through which elites restrict the wider population's access to resources and opportunities, and the role of exclusive organisations in maintaining cohesion among a select few, have important implications for social inequalities. Drawing on 41 semi‐structured interviews with wealthy members of the business class living in and around Manchester in northern England (21 of whom were members of a CEO peer group), I analyse how three social processes—homophily, structured reciprocity and therapeutic cultural resources –make and legitimate cohesion between members, as well as instances of when cohesion fails. In doing so, I explore how therapy culture has travelled upwards, to the executive and owning class, through CEO peer groups. I make the case that CEO peer groups represent a fruitful entry point into wider debates about class formation for the contemporary business class in the UK and, given their global scope, more broadly.

## 1 Introduction

In the current context of extreme economic inequality and rising concentrations of income and wealth at the top (Chancel et al. 2022), the social processes through which elites restrict the wider population's access to resources and opportunities, and the role of exclusive organisations in maintaining cohesion among a select few, have important implications for social inequalities (Scott 2008, 35). In fact, for Savage (2014, 603), ‘the kinds of closure and social and cultural elitism which might now characterize the very highest levels of the social structure’ are ‘arguably the fundamental sociological questions of our age’. Drawing on 41 semi‐structured interviews with wealthy members of the business class living in and around Manchester in northern England (21 of whom were members of a CEO peer group), I make the case that these represent a fruitful entry point into wider debates about class formation for the contemporary business class in the UK and, given their global scope, more broadly.

The term ‘emotional capitalism’ was coined by Illouz (2008, 18) to describe, ‘a broad, sweeping movement … in which emotional life—especially that of the middle classes—follows the logic of economic relations and exchange’. This dual process where emotional and economic relationships influence and reshape one another has seen taken for granted cultural binaries, such as masculine/feminine, private/public or emotional/rational, destabilised. For instance, as workplaces increasingly rely on emotional skills, self‐expression and intimacy, she outlines how a feminine emotional style has become a form of symbolic capital among managers within a formerly masculine corporate culture (Illouz 2008, 231). For Illouz (2008), ‘the therapeutic’ can be understood as a set of cultural resources that lets people accomplish things. Beyond the middle classes, others have mapped how intellectual fluency in therapeutic narratives acts as mark of distinction for upper class, liberal parents (Ramos‐Zayas 2023) or a productive emotional strategy for managing difficult circumstances for working class women (McLeod and Wright 2009). Up to now, however, there has been very little consideration of the impact of the therapeutic turn further upstream in the corporate environment ‐ for the C‐suite and owners of significant business enterprises.

I call the CEO peer group in this article the Organisation for Business Peers (or OBP). Founded in the mid‐twentieth century in the USA, OBP claim to have a global membership in the tens of thousands who lead businesses and organisations worth trillions of US dollars in annual revenue. As with other CEO peer organisations, OBP requires that members meet minimum thresholds of seniority and business size. To join OBP you are required to have a leadership role, such as CEO or equivalent. You are also required to have a minimum number of employees, which varies to accommodate smaller companies with higher employee compensation, and a minimum revenue or enterprise value, which varies depending on the type of enterprise. OBP is one of the more exclusive organisations facilitating CEO peer groups. The entry requirements position OBP members as part of a ‘business class’ in as far as they occupy a shared ‘capitalist class situation’ (Scott 1991, 65), that is they all owe their life chances to property or control in the sphere of large‐scale capital, and are interconnected through circulation and interaction (Scott 1997, 276–280). The organisation provides a way for different kinds of members of the business class to meet, whether the founders of their business, the heirs of family businesses or professional managers. Membership of OBP is male dominated. According to internal documents circulated by OBP, 12% of members were female in 2025.

In terms of what membership offers, OBP runs regular educational, social and networking events open to the entire membership of regional chapters, which often centre on an invited speaker—say, from the arts, business, politics, science or sports. Members can bring their spouses to these chapter‐wide gatherings. In addition, members can join international networks focused on specialist interests—for example, business deals, parenting or vintage cars ‐ attend global conferences and receive regular communication from the organisation.

But at the heart of OBP are peer groups ‐ or forums ‐ of non‐competing business leaders who come together in a confidential environment to discuss business, family and personal issues. Forums are usually made up of six to eight members. Rather than paid professionals, OBP forums are peer moderated by members themselves. OBP chapters do not have physical sites, so forum and chapter events rotate between private spaces, such as hotels, office spaces or members' homes. Being part of a forum represents a significant time commitment. Members usually meet once a month for a period of 4 hours over most of the year. They also attend an annual retreat with their fellow forum members lasting for one or several days. The same forum can run for years or even decades. I find it useful to think of forums as a group coaching session. During forum, members engage in carefully structured conversations, which can range from business topics, such as the threat of a hostile takeover, comparing interest rates on a loan or minimising tax when selling a company, to more personal concerns, such as marriage proposals, their relationship with their father or how to manage a spendthrift heir. Forums aim to create a ‘holding environment’ for their members (Kahn 2001; Petriglieri and Petriglieri 2010), where they can acquire self‐knowledge, build meaningful connections with other members and access the collective experiences of their peers. The forum method has been scaled successfully and forms a core element of OBP's approach and success. It is also the focus of this paper.

The first empirical section, ‘homophily’, examines the preference for similarity in terms of shared seniority, wealth and an affinity for group coaching. The second empirical section, ‘structured reciprocity’, explores the role of forum protocols to ensure a sense of mutual benefit between members and the significant business support members were able to access from their peers once the risk of competitive rivalry has been removed. The third empirical section, ‘therapeutic cultural resources’, analyses the use of training and communication protocols to create an environment where members felt able to be open and vulnerable without fear of judgement. The fourth and final empirical section, ‘when cohesion fails’, confirms the importance of the above social processes for cohesion through exploring when membership was refused or failed to appeal.

## 2 Elite Cohesion and Therapy Culture

The concept of a close‐knit ‘power elite’ with a shared educational background, social clubs and familial ties has been highly influential (Mills 2000 [1956], 281). This shared experience matters in so far as it transforms elites from occupants of a purely formal category into a ‘cohesive and solidaristic social group’ (Scott 2008, 34). Cohesion between British business elites has traditionally been understood as driven by exclusive education systems, private member's clubs and corporate interlocks. However, the significance of each of these institutions has been steadily eroding since the late 1970s (Reeves et al. 2017; see also CSM 2014; Davis 2017, Scott 2003; Maclean et al. 2006; Moran 2008; Bond et al. 2010; Cronin 2012).

More recently, the Millsian ‘power elite’ approach has been criticised for applying a theoretical approach developed for mid‐twentieth century USA to very different contemporary contexts (Savage forthcoming). For a more fragmented elite operating in a globalised and financialised context, Savage and Williams (2008, 15) argued a couple of decades ago that we need an updated approach, beyond traditional ‘old boy networks’ or ‘inner circles’. More recently, focusing on the UK, for Jones's (2015), the British elite are no longer held together by social clubs or shared backgrounds, but by a loose affiliation to neo‐liberal assumptions. For a more professionalised and liquid British business elite, Davis (2017) has documented the rising significance of professional business schools and regular, semiformal types of social interaction, such as the World Economic Forum or networking events hosted by intermediary firms like McKinsey (p. 242, 235). What has been missed so far is the role of CEO peer organisations in making and legitimating cohesion among members of the business class.^1^

One way to understand CEO peer groups is in relation to other clubs that drive cohesion for the business class. London's old‐school private members' clubs operating since the nineteenth century provide one important origin story. Private members' clubs have long offered an informal mechanism of elite integration and cohesion; and a badge of status and acceptance for a diffuse upper class (Milne‐Smith 2011; Scott 1991; Mills 2000 [1956]; Baltzell 1958; Domhoff 2005). From their inception, information gathered in clubs was considered sacred. In fact, this privileged information and code of ethics as to how to protect that knowledge is argued to have played a key role in creating upper‐class community (Milne‐Smith 2011, 98). As Simmel (1906) put it in his classic work on the theme, ‘[s]ecrecy and pretence of secrecy are means of building higher the walls of separation, and therein a reinforcement of the aristocratic nature of the group’ (p. 486–487). American service clubs, such as Rotary or Lions, also provide useful context to understand CEO peer organisations. From the early twentieth century, these have allowed middle and upper‐middle class men to develop business contacts more instrumentally than traditional members' clubs (Wikle 2004; Cousin and Chauvin 2014) and, similarly, globalised rapidly in a context of US hegemony (Charles 1993; Goff 2021).

Another, perhaps more surprising, origin story is the Human Potential Movement.^2^ This loosely organised social movement is broadly aligned around a belief that self‐actualisation is best facilitated when like‐minded individuals get‐together and mutually seek personal growth (Spence 2007, 4). It centres free expression and was closely aligned with humanistic psychology (Howard 1970). Key institutions and methods from this movement can be directly linked to the early development of executive peer advisory groups—including experimentation with T‐groups in the late 1950s and 1960s and the Esalen Institute in the 1980s (McNees 2000). Today, such experiential, group learning methods continue to shape leadership training and approaches to productivity more broadly, notably for Ivy League business schools (Stanford 2025) and tech corporations (Chen 2022) in Silicon Valley.

In their now classic work mapping contemporary capitalism, ‘The new spirit of capitalism’ Boltanski and Chiapello's (1999/2018) idea of ‘the artistic critique’ helps clarify this corporate use of counter‐cultural ideas and practices. The artistic critique explains that anti‐capitalist counter‐culturalists, artists and scholars had long criticised industrial capitalism for constraining creativity and autonomy and for creating alienating work relations (p. 343). But capitalism was able to appropriate this critique and, in doing so, rejuvenate itself, ushering in a new world of work based on creativity, networking, flexibility and self‐control. These ideas have been explored through the concept of the entrepreneurial self (e.g., Rose 1999, Bröckling 2015). Closely related to this shift to neoliberal capitalism is the rising influence of therapy culture. Therapy culture can be defined broadly as referring to ‘eclectic’ (Illouz 2008, 12) expressions of ‘psy’ discourse (Rose 1996) in expert and popular knowledge, ranging from the more formal therapist‐patient relationships to the now widespread faith in self‐examination, positive thinking and open communication to alleviate life's problems (Madsen 2014; Aubrey and Travis 2015). In this article, I explore how therapy culture has travelled upwards, to the executive and owning class, through CEO peer groups.

## 3 Methods

As part of a broader 3‐year qualitative study examining concentrations of wealth in north‐west England, I conducted 41 semi‐structured interviews with the wealthy business class living in and around Manchester, England between May 2018 and July 2021. The sample list of interviewees was drawn from national and local Rich Lists and the commercial data provider Wealth X. During the interviews, I initially asked whether interviewees were members of any clubs, societies or organisations for business leaders. In and around Manchester, there are a small number of private members' clubs and VIP lounges, but at the time these appeared to primarily cater to professionals in the service class (such as accountants or lawyers) and local celebrities (such as footballers and reality TV stars), rather than leading members of the business class themselves. Once I learnt about the organisation during my first interview, I then also asked more specifically about membership of OBP. Twenty‐one interviewees were OBP members. Of the 20 non‐members I interviewed, most were aware of it but had not been invited to join, a small number of them had been invited but turned membership down and a smaller number again had not heard of it.^3^ While these are not the focus of this article, in addition to interviewing members of the business class, I interviewed 61 professional intermediaries who provided services for them in the region, such as real estate agents and wealth managers, many of whom knew about OBP. I also interviewed one of the certified facilitators who operate globally training OBP members in the communication styles and protocol required to participate in forum.

Of the 21 interviewees who were OBP members, 18 were men and three were women. They ranged in age from 35 to 80, with a median age of 58. Where possible,^4^ the net worth of members was ascertained from access to the wealth intelligence dataset Wealth‐X and publicly available data online, and ranged from £55 million to over £1 billion. Their companies were in financial services, health, manufacturing, real estate, retail and technology sectors. The companies that interviewees led ranged from household names listed in the FTSE 100 to medium‐sized enterprises with little public profile. The interviewees had held their leadership position for years, if not decades, so were not characterised by the short‐term contracts of a liquid managerial elite (Davis 2017). Fifteen individuals were the founders of the business where they generated the majority of their wealth and six were inheritors of a family business.^5^ Of those OBP members who were founders, one was formerly a professional manager, but he later started his own business, partly following the encouragement of other OBP members.

In terms of school, one member attended a Clarendon school,^6^ 12 attended other private schools and nine attended non‐fee‐paying grammar or comprehensive schools,^7^ which means a notably higher percentage attended fee‐paying schools compared to national studies of British business leaders (Maclean et al. 2006; CSM 2014; Davis 2017). No particular school appeared consistently across interviewees. Four went to Oxbridge, eight attended Russell Group universities,^8^ one attended a Plate Glass university,^9^ one attended a Polytechnic university^10^ and seven did not attend university. Four had attended the same Russell Group University in the region, but would not have been there at the same time. All of the members interviewed were from the region or had accumulated their wealth there. Nineteen were white and two had South Asian heritage. A member I interviewed said that the region's chapter has 40–60 members, 7%–15% of whom are women. While I was not able to verify this percentage, it matches my own observations among my interviewees. The selection criteria for my original study—the wealthiest business elites in the region—means I was more likely to recruit older members and business owners. Despite these limitations, given that OBP membership requirements, training and protocol are (at least intended to be) standardised, the social and cultural processes documented in this article are likely relevant beyond this case study.

Participants have been given pseudonyms and identifying characteristics, such as a location, brand name, business sector or even gender, have been altered to preserve anonymity, with every effort made to make sure this alteration does not affect the interpretation of the associated quote. Tolich (2004) makes the distinction between external and internal confidentiality. Researchers have to ensure that people both external and internal to a group cannot identify others with certainty. In order to ensure internal confidentiality, in cases where participants' characteristics or actions may make them identifiable to other members, I have taken extra steps to disguise their identity. OBP is a pseudonym, and publically available data points about the organisation have been included in broad‐strokes to prevent direct identification.

Finally, I also compiled and analysed an archive of 42 publically available documents published by the OBP, or OBP members, about the organisation, such as forum guidebooks, which provide insight into how the organisation presents itself publicly and how forum operates, albeit in an idealised form. These are drawn on in the analysis but are not cited to avoid identifying the organisation.

Using a thematic analysis approach, I started analysis by first familiarising myself with the interview transcripts and archival materials, then coding them to generate initial themes. Next, I conducted another round of coding to develop and review these initial themes, while continuously seeking out disconfirming evidence, before eventually refining, defining and naming the themes outlined next in an iterative process (Braun et al. 2016). In the following, I introduce and analyse three social processes—each influenced by therapy culture ‐ that I argue make and legitimate cohesion in CEO peer groups.

### 3.1 Homophily: ‘A Board of Non‐Executive Directors’

It’s almost like a board of non‐executive directors. These people are all experienced. All have their own contacts … [and] they’ve no axe to grind, so they’re not ‘Yes men’. They’ll say, ‘What [are] you talking about? That’s stupid.’^11^

(Jim)

Homophily ‐ a preference for similarity ‐ is perhaps not a surprising finding in an exclusive members' club, but the particular form this takes for CEO peer groups is, I think, interesting. As mentioned, prospective members have to meet ‘hard’ criteria to join in terms of holding a leadership role in a significant business enterprise. The different thresholds for net revenue and number of staff try to accommodate different types of sectors at different stages of growth ‐ think of the difference between a tech‐start up and a fourth generation retail company or an international hedge fund and a national media agency. Prospective members also have to meet ‘soft’ criteria in terms of their likely commitment and social fit.

OBP arrived in northern England in the 1980s. The founder had been a member in London, where he was encouraged to launch a chapter in northern England. In preparation, he asked his lawyer and accountant to make a list of local successful business leaders, and then invited prospective members for lunch at his company. If this initial meeting went well, he then invited them to bring their wife to dinner with him and his wife. (At the time, all of the members were men). Today, members can join OBP through referral or apply to join without one. Every chapter has its own recruitment process, but in all cases prospective members (and in some cases their spouse), are screened by current members in terms of whether they meet the required criteria, how well they would integrate and whether they have a good reputation professionally and personally.

As Jim's (late 60s) quote opening this section shows, forums meet their members' desire to benefit from the collective experience of other senior business leaders. The phrase, ‘it's lonely at the top’ came up over and over again during interviews. Forums are designed to ease that isolation by bringing together senior business executives in a context of guided intimacy. Given this professional isolation, members' reflected on the value of receiving frank and impartial feedback from their peers. Returning to Jim, he summarised this isolation, and the benefits of forum:

Unlike when you're running a company and you've got your management team, your board of directors, if you have a really stupid idea, they might not tell you that it’s a stupid idea. Whereas, in your forum, they will most certainly tell you it’s a stupid idea, because they’ve not got to be careful what they say. … You can go along to your business advisors, your solicitor, your accountant but you’re paying them a fee, so they’ll be careful what they say to you as well. You get great unbiased opinions from your fellow OBP members. If you face a problem, it’s just about certain at least one of them will have faced that problem themselves and can give you that unbiased advice. It’s just the best influence you could ever hope for.

This view of the member as isolated was repeated as common‐sense across most interviewees, and provided a justification for the creation of exclusive peer groups to address that separation.

After seniority, the second shared characteristic of members is wealth. Forum enables a specific kind of class‐based relational vulnerability: the ability to talk with other wealthy people and thus to manage shared anxieties attached to wealth (Sherman 2017). For instance, Sam (early 60s) explained, ‘If I go back to my best man who works in York, he isn't interested in my problem of inheritance, is he? That's an alienating problem to discuss with someone who doesn't have that problem.’ Sam went on to compare OBP to Alcoholics Anonymous, an addiction recovery programme:

If you’re alcoholic and you want to get advice, go and live and breathe with other alcoholics who have recovered. If you’re a business leader that’s accumulated some wealth for you and your family and you’re a bit anxious about it, talk to other people who are managing that problem. So OBP is a group of peers who have got likeminded challenges, whether it’s rearing children or running generational transitions in family businesses. It thrives because of the likeminded conditions you’re bringing together. Like any group does, whether it’s a church or whatever.

In this framing, membership of OBP offers a community of peers who share and understand the unique social position of extreme wealth. Rather than an emphasis on exclusivity or prestige, forums are portrayed here as offering practical support groups for ‘peers who have got likeminded challenges’, ‘likeminded conditions’ and the ‘problem’ of inheritance, with wealth and economic power presented in either neutral or negative language.

During our interviews, several members reflected on what they perceived as a necessary willingness to be vulnerable and to learn from others as desirable traits to integrate successfully into a forum. For instance, when I asked about the training required to join, Alice (40s) said that the vetting of new members was equally as important as training ‐ ‘is this person willing to be vulnerable? Is this person willing to listen to other people and to try and bring everything they can to support the other person?’ When I asked if there was such a thing as a typical member, Sam emphasised humility,

I think if you were an exceptionally self‐centred business leader OBP probably wouldn’t be very attractive to you. … I think you’ve got to have some sort of desire to learn from other people and develop a certain humility. To learn from others does require a certain humility.

The economic and status thresholds to join ensure members have relevant experiences to share during forum, but alongside this ‘hard’ criteria prospective members were also screened for a perceived openness to group coaching and its ethics of self‐disclosure and self‐realisation.

### 3.2 Structured Reciprocity: ‘A Self‐Help Club’

It was almost like a self‐help club really, in a sense, but for entrepreneurs and senior business people.

(Mike)

As much as self‐help has come to imply a focus on the individual, it also points to the idea of mutual support. A self‐help club is a gathering of people who come together to address a shared problem. As recently as the 1970s, ‘self‐help’ referred not to individual self‐improvement practices but to cooperative efforts for mutually improved conditions on the part of a community of peers (McGee 2005, 7). While OBP steers clear of supporting any political or social cause, forums are cooperative efforts, rather than an individual undertaking. For the business class, this cooperation can pose challenges. Marx (1894/1959, 253) famously described capitalists as ‘hostile brothers’ ‐ with shared class interests but competitive commercial interests. OBP certainly has a lot of rules to ensure members of the business class socialise successfully. Here, I use structured reciprocity to describe the protocols put in place to make sure forums are mutually beneficial.

Crucially, there are rules in place to overcome potential commercial rivalry between members. Groups are organised along geographical lines that purposefully separate members who operate in the same industry. If a member moves into an industry in which an existing member is already doing business, thus making the two members competitors, the newest arrival to that industry is expected to leave the group. Protocols emphasise that members are also advised to avoid going into business together, as the commercial dimension to their relationship could limit their ability to be honest and vulnerable.^12^ Philip explained why this organisation of membership was beneficial:

Why would you tell a competitor how you did something? But if you’re going to a fellow business leader that’s got a retail business … he or she would be very happy to share their learnings because they know that it’s not going to affect their business. You’re a manufacturer, so why not?

Forums are organised so that members can support their fellow members' commercial success without the risk of undermining their own.

In addition to minimising the risk of commercial rivalry, a successful forum requires mutual commitment from members. For a high‐powered membership with multiple demands on their time, the monthly meetings demand significant and sustained commitment. Without rules to ensure mutual commitment, members risk investing time and energy in others who do not repay the favour. Mark (early 60s) thus differentiated OBP from other members' clubs:

It’s not like being a member of a golf club where you can turn up when you feel like it. Generally, the groups work best when people participate and we want people to feel that it’s a two‐way thing. Most of the people in the organisation are running large businesses and if they’re going to make the commitment, they want other people who are in the same groups as them making the same commitment. If you don’t, then you’re kind of taken to task over it and if you continue not to be committed then, in very, very rare cases, they say, ‘Look this isn’t for you, it’s not working.’

A round of golf with other business executives represents the casual side of elite sociability, whereas forum is highly structured. Forum protocol can vary and, and there is a strong emphasis on confidentiality, so I was unable to observe a forum directly. However, published guidance provides a useful insight. This guidance stipulates that members must arrive on time (or risk being fined if they are late); follow attendance norms (they cannot miss too many sessions); give their full attention during the session (phones are put on silent and laptops are put away); and are allocated equal time to speak. OBP guidelines state that these protocols are designed to support equality and inclusion between members. Once someone has met the requirements to join, forum protocol aims to cultivate an atmosphere of horizontality between members, no matter their different status outside of the group.

Finally, once the various hurdles of cooperation have been overcome, it is worth illustrating some of the concrete support members offered one another. Alice provided two examples of support in business strategy she received from other members. First, when she had to deal with a regulatory authority in the UK, another member rang saying they had just been through the process, ‘come in for an hour and I'll explain to you what I found, who we found helpful, mistakes not to make, so forth’ Second, she told a story about the practical and emotional support she received when she called an emergency meeting with the members of her forum after a business rival used aggressive tactics:

I just sent a message to my forum and asked, ‘please could everyone meet at my house at 7pm tonight. I’ll bring pizza and we can work out what I do with this next.’ And every single one of them was there at 7pm with ideas. We sat with a white board in my kitchen. I let out the bits that I was scared about and I let out that I was angry that it had been taken out of my control.

In another example, Ben (late 40s) explained that his forum was in touch,

all the time. If I showed you my Whatsapp there’s always stuff. Does anyone know this person? What do you think about this? Here’s an idea I’m thinking about. All the time.

Another member Philip (early 50s) explained that before and during the process of listing his company's shares publicly for the first time,

I was very careful to make sure I went round a number of OBPers who had similarly IPO’d^13^ their business and asked them for advice on how to do so. … I was able to pick the brains of people who … had listed their business, who had created a national, well‐known household name.

These examples point to some of the valuable expertise and networks membership opens up, as well as an informal support network that extends outside the officially curated space of forum and chapter events.

### 3.3 Therapeutic Cultural Resources: ‘Group Therapy for Chief Execs’

This is not an official description but for me OBP is group therapy for chief execs. … You’re there for people’s ups, you’re there for people’s downs. It’s not about backslapping and just making sure you all earn more money. It’s a little bit more real world and supportive than that, is my experience.

(Alice)

OBP guidelines emphasise that members' personal and family lives cannot be separated from their business lives. Forums draw on therapeutic cultural resources to create a space where members are rewarded for interpersonal honesty, disclosure of self‐doubts and perceived weaknesses in all aspects of their lives. Drawing on the accounts of participants and published materials, a typical forum might start with a check‐in, followed by a trust‐building exercise, then updates from every member, before focusing in detail on one or two members' presentation of a problem or opportunity in their professional or personal life. Sam explained the importance of following this meeting structure:

You had a lousy journey to the meeting or someone’s ill in hospital or some business deal has collapsed, it’s good to say that at the beginning of the meeting because that’s going to affect your mental presence, isn’t it? … Then there’ll be a trust‐builder process, which will ask a general question like, ‘Reflect back on what ways you think your mother shaped who you are today?’… When you ask trust‐builder questions, which provoke vulnerability, it starts to put you in the right zone for the rest of the meeting. Then we’ll do a process of quick updates, what’s gone on in my business, family, personal life since we last met.

The updates and trust building exercises are designed to encourage members to open up and share with the group. The achievement of vulnerability during a meeting is a stated goal, with associated protocols and training.

While masculinities in corporate workplaces may be changing, management and business scholars still observe that dominant expectations of senior leadership emphasise conventional masculine norms of control, emotional restraint and self‐reliance (Hay 2013, 512; Corlett et al. 2021). This context may explain why Nick emphasised the importance of training to be able to display vulnerability in front of other business leaders:

…you’ve got to create an environment of shared vulnerability, which in itself requires a lot of training … in what could be quite an alpha environment … [so] I can take issues and feel like I'm not going to get judged about them … whether it be something in your marriage, something with your kids or something in your business.

For Nick, ‘the absolutely confidential nature of anything that is discussed’, helps make forum ‘an incredibly safe space to have those conversations.’ OBP guidelines encourage forums to remind members about confidentiality at the start and end of every meeting. On top of this, groups will periodically review a confidentiality case study to further embed its importance. As Nick illustrates, this confidentiality is justified through the therapeutic ambition of creating a ‘safe space’ where members can discuss personal and business issues openly.

While Nick's reference ‘alpha’ does not inherently imply male, it does infer traits stereotypically coded as masculine, such as dominance, competitiveness and strength. It is perhaps notable that none of the three female interviewees mentioned the difficulty or novelty of emotional vulnerability during forum. Reflecting on OBP more broadly, Ryan (late 30s) emphasised the gendered benefits of membership,

I think the biggest takeaway from it is the ability for leaders, particularly men, to go into emotional vulnerability … opening up that space has got to be a societal benefit.

While it is beyond the scope of this article to examine how CEO peer groups shape members' business leadership, the gendered dimension sparks interesting questions. We might think of forums as offering an acceptable route for male CEOs to ask for help, express vulnerability and build (different kinds of) intimacy with other men. In the private space of forum, displays of emotional vulnerability (and, as we have seen earlier, empathy and humility) are prized among members, traits that are more conventionally coded as feminine. Beyond the middle classes (Illouz 2008), this finding points towards how feminized versions of masculinity are valuable in the workplace even for those at the very top of corporations. An equally convincing argument, however, is that the private adoption of traditionally feminine traits in forum supports the continued public performance of traditionally masculine norms and expectations.

The therapeutic cultural resources permeating interviewees' accounts of their membership can be traced back to the training material produced by OBP. For instance, when listening to other members, training material advises members to focus on the speaker's feelings and the feelings provoked in themselves, to use active listening, to suspend judgement, to avoid the word ‘should’, to be specific rather than to generalise and to respond using ‘I’ statements that speak from their own experience. Conversely, when speaking, members are trained how to appropriately self‐disclose and to elicit and receive useful feedback through active, non‐defencive listening. Forum protocols are professionally supported and disseminated across the membership. For instance, Alice explained that an external, paid, OBP‐certified forum facilitator came in every 12 months to remind members of the correct protocols during a forum retreat, perhaps running through role‐plays to work on issues facing the group. She went on, ‘they might sometimes say, “hang on a minute, this is getting a bit advise‐y and you don't do advice.”’ Instead, members would be encouraged to say, ‘“an experience I have had”, “an emotion this brings up for me”, something like that… “you should do this” isn't a conversation that should ever be had.’ Besides external facilitators, OBP guidelines advise that a member of each forum is allocated the task of making sure other members observe forum protocols. Participants' accounts, however, reveal that they did not always meet these standards, as with Jim's earlier comment on his forum peer telling him an idea was ‘stupid’; I explore some of this discrepancy further in the next section.

### 3.4 When Cohesion Fails

We can further infer that peer groups rely on homophily, structured reciprocity and therapeutic cultural resources to operate smoothly from the instances when interviewees found their membership less valuable, left or declined an invitation to join. Reflecting on his membership, Mike said, ‘In the earlier days it was very helpful in that you could share issues with likeminded people.’ However, he found his membership less useful after his company started to experience rapid growth, explaining that he felt he outgrew his forum. While membership is premised on shared business success, there are gradations within that, with some members running significantly larger enterprises than others. His company's growth meant he no longer felt his forum group had relevant experiences to share. When I asked about OBP, Paul (50s) said, ‘a lot of these groups pitch themselves as, ‘’It's lonely at the top, you can never speak your mind, so get together with this group of similar individuals in confidence and share all your thoughts,’’ but I've never felt that’. He positioned himself, in contrast, as talking, ‘very openly to the people within [the company] … if we're doing well we share it, if we're not doing so well we share it’ and ‘a small group of friends’. In his case, accessing peer support from other senior leaders of large business enterprises to address a sense of isolation did not appeal.

For others, the artificiality of the group's sociability was off‐putting. Max (late 40s) had been approached to join, but had declined, explaining that, ‘sharing my business strategies and deepest thoughts and chatting to semi‐strangers, that's not what I do.’ Max preferred to spend his weekends with family and friends that he had known for many years. He went on, ‘I don't want to do it with a load of business people.’ While he was supportive of the concept, saying, ‘I absolutely understand it works for some people and good luck to them,’ membership was not attractive for Max. He positioned himself as a ‘maverick’, who already had a broad network whom he could call upon for business support, and did not want to join a group with strict rules. ‘I'm not good at being in a club,’ he said, ‘it's just not me.’

In another instance, David (late 40s) had been a member but had since left. He said, ‘it wasn't for me. There are a lot of rules associated with it.’ He accepted that the forum protocols were understandable as a strategy to level the status between members, as ‘obviously there are some massive egos in the room so it's all about following the rules and the rules apply to everybody’. However, despite this structure, he went on, ‘I always felt like there was an inner circle and a clique‐y circle and a ‘‘this’’ circle and a ‘‘that’’ circle.’ While acknowledging, ‘as a network it's unbelievable,’ David had decided, ‘it wasn't for me. I just can't be doing with all the egos and the drama’. In this example, the organisation's aspiration to create an inclusive atmosphere of social horizontality between members was not realised. David also mentioned repeated arguments with other members about how little they paid their staff, revealing some of the fault lines in ideological attitudes between members,

I think there are some incredibly wealthy people in our region who have minimum wage workforce who don’t need to have a minimum wage workforce that can afford to pay better and just choose not to ‘cos it’s all about profit and I just think that’s wrong. […] And having had one too many arguments with members of OBP about this subject, I decided it wasn’t the group for me.

While David shared a class position and openness to group coaching, he felt his values were too different from some of the members. In sum, when a business leader is not attracted to, or convinced by, the homophily, structured reciprocity and therapeutic cultural resources that structure forum in OBP, cohesion fails.^14^

## 4 Conclusion

CEO peer groups justify themselves through the idea that its ‘lonely at the top’. Wealth and power can elevate people into an isolated sphere of existence. As we saw in the section on ‘homophily’, this sense of isolation—driven by the idea that others want something from you or, at best, do not understand you—justifies a particular solution: to socialise in a bubble with other wealthy members of the business class. In creating an exclusive social space as a solution to isolation from wider society, however, CEO peer groups only serve to reinforce that separation. Beyond their shared class position, homophily is driven by a particular idea of business leaders as lonely and as in need of community between peers. CEO peer groups aim to recruit new members who share this psychological problem of isolation. The section ‘when cohesion fails’ showed that this problem did not resonate with all members of the business class.

CEO peer groups reveal without a doubt that therapy culture has reached the top tiers of the business class. In fact, their version of self‐help is in many ways more cooperative and less individualistic than the mainstream (McGee 2005). The section ‘structured reciprocity’ explored the particular challenges forum poses as a cooperative endeavour for the business class, and how they overcome these problems to achieve significant mutual benefit. In this way, counter‐cultural ideas rooted in the human potential movement and experiential learning through groups have been adopted and reworked by the business class. Finally, the section ‘therapeutic cultural resources’ identified the protocols, training and guidelines required to create a space where therapeutic and coaching norms—such as expressing emotional vulnerability—are aspired towards and realised. Alongside indicating economic and social success, membership of CEO peer points towards the pursuit of a particular kind of cultural capital among some members of the business class ‐ fluency in therapy culture. Returning to Illouz (2008), if ‘therapy culture’ lets people accomplish things, in the case of CEO peer groups, its role might be usefully understood as an important and so far unexplored mechanism for how members of the business class forge solidarity with one another. Given this argument, a better understanding of who the membership of global CEO peer groups are, how they use their membership and how this might vary across different geographies has important implications for our understanding of how today's business class are socialised, how they connect with one another and how they reproduce themselves.

There is a risk that my emphasis on the centrality of therapy culture for CEO peer groups itself becomes part of their legitimation. It is important to emphasise that such groups ultimately lock in wealth, advantage and connections between their (predominantly male) class‐advantaged members. Private and exclusive spaces for elites have come under sustained criticism by the media and the public (Heemskerk 2007). Against this backdrop, therapy culture provides a thoroughly modern form of legitimation for forms of exclusive elite sociability that may, nevertheless, prove hard to reconcile with popular support for greater transparency among the wealthy and powerful.

## Conflicts of Interest

The author declares no conflicts of interest.

## Data Availability

The data for this project is not publicly available.

## Endnotes

^1^
What little academic research I have found on CEO peer organisations amounts to unpublished doctoral theses exploring their efficacy for members (Olmsted 2019; Feghali 2022), a survey comparing emotional intelligence among members and non‐members (Stein et al. 2009), and interview‐based studies exploring members' retirement plans (Sonnenfeld 1991) and the role of accountability for achieving goals among small business owners (Zuckerman and Sgourev 2006).

^2^
In a less counter‐culture reading, the best‐selling self‐help book, ‘Think and Grow Rich’, traces the lineage of CEO peer groups to Benjamin Franklin's mutual self‐improvement ‘Junto Club’ in the eighteen‐century (Hill 1937/2004).

^3^
While there is not space to explore the characteristics of participants who are not members in full, I will briefly note that they are slightly older (with a median age of 63), but, otherwise, their wealth, education and whether they are a founder or heir of their business is not substantially different from participants who are members. However, the small number of interviewees who had not heard of OBP were notable for having a low public profile.

^4^
Two of the members' wealth was not publicly available information, but they were included in the study to increase the number of female participants and members of OBP, respectively. They also both met the threshold to join OBP, which positions them as running a significant business enterprise.

^5^
A minority of ‘founders’ had scaled what was a considerably smaller family business—in which case, the term ‘venture owners’ may be more appropriate (Stamm 2023).

^6^
A group of nine leading independent schools.

^7^
However, of these nine interviewees, two received scholarships to attend a direct grant grammar school, which blur the line between fee‐paying and non‐fee‐paying schools (Gamsu 2020), and could be interpreted to mean an even higher percentage of participants went to private schools.

^8^
A self‐selecting association of research‐intensive universities with high academic entry requirements.

^9^
Plate‐glass refers to universities that emerged in the 1950s and 1960s, which are often in a modernist architectural style, and feature large sheets of glass.

^10^
A polytechnic was a higher education institute that specialised in vocational or technical subjects.

^11^
It is worth noting that Jim's quote contradicts the therapeutic cultural resources explored later, pointing to the possible distance between how forums operate in theory and in practice, but also possibly generational differences in the culture of forums as he is an older, long‐standing member.

^12^
However, membership does result in significant flows of investment, as well as philanthropic donations and personal favours. As one family office professional said of the OBP, ‘pretty much every successful person in the north is a member of it. They're super supportive of each other. They share all sorts of business advice, personal advice, philanthropy advice, investment opportunities’.

^13^
An IPO, or initial public offering, refers to when a privately owned company lists its shares on a stock exchange.

^14^
OBP is not unaware of these potential pitfalls, and it publishes advice on issues such as forums going stale, managing friendships between members so as not to exclude others during forum meetings and dealing with deeply different values between members.
